# Combination of Cordycepin and Apatinib Synergistically Inhibits NSCLC Cells by Down-Regulating VEGF/PI3K/Akt Signaling Pathway

**DOI:** 10.3389/fonc.2020.01732

**Published:** 2020-09-07

**Authors:** Xiaozhong Liao, Lanting Tao, Wei Guo, Zhuo-Xun Wu, Haiyan Du, Jing Wang, Jue Zhang, Hanrui Chen, Zhe-Sheng Chen, Lizhu Lin, Lingling Sun

**Affiliations:** ^1^Department of Oncology, The First Affiliated Hospital of Guangzhou University of Chinese Medicine, Guangzhou, China; ^2^Department of Pharmaceutical Sciences, College of Pharmacy and Health Sciences, St. John’s University, Queens, NY, United States; ^3^Department of Biochemistry, Guangzhou University of Chinese Medicine, Guangzhou, China

**Keywords:** cordycepin, apatinib, synergistic effect, A549, PC9, VEGF/PI3K/Akt signal pathway

## Abstract

**Background:**

The application of apatinib is immensely limited by its acquired drug resistance. This research investigates whether cordycepin, a component from Cordyceps could synergize with apatinib to improve its anticancer effect on non-small cell lung cancer (NSCLC) cells.

**Methods:**

The NSCLC cell lines A549, PC9, and H1993, and human bronchial epithelial (HBE) cell line Bears-2B were used in this study. Cell counting kit 8, colony formation assays, wound healing assay, transwell assay, and flow cytometry analysis were performed to assess the cell viability, the migration ability, and invasion ability of the cells. Kyoto encyclopedia of genes and genomes (KEGG), western blotting and molecular docking was applied to analyze the possible pathways affected by cordycepin.

**Results:**

The combination of cordycepin and apatinib in a ratio of 5:1 synergistically reduced proliferation of NSCLC cells, inhibited cell migration and invasion, increased cell apoptosis by altering cell cycle in NSCLC A549 and PC9 cells. The VEGF/PI3K/Akt pathway was inhibited after treatment with cordycepin and apatinib.

**Conclusion:**

Our findings demonstrated that the combination of cordycepin and apatinib has synergistically anticancer effect on NSCLC cells by down-regulating VEGF/PI3K/Akt signaling pathway. This result indicated that cordycepin and apatinib could be a promising drug combination against NSCLC.

## Introduction

Lung cancer is one of the cancers with high morbidity and mortality, which consists of more than 50 histological subtypes (1, 2). Non-small cell lung cancer (NSCLC) is the dominant type, accounting for about 80% of lung cancers (3). The combination of conventional platinum chemotherapy and radiotherapy is the standard therapy for locally advanced unresectable NSCLC (4). Despite that surgery, chemotherapy and radiotherapy increases the survival rate, the significant side effects induced by such treatments seriously impair the prognosis of cancer patients. There is a growing demand for safer and more efficient treatments (4, 5).

Apatinib, a new selective vascular endothelial growth factor receptor-2 (VEGFR-2) tyrosine kinase inhibitor (Supplementary Figure S1), plays a significant role in the therapy of advanced metastatic gastric cancer. It was confirmed that apatinib can be used as subsequent-line treatment for other advanced or metastatic solid tumors, such as NSCLC, breast cancer and hepatocellular carcinoma (HCC) (6). An increasing number of clinical trials have reported that apatinib has potential benefits in the treatment of patients with advanced NSCLC (7–9). And apatinib was demonstrated to reverses multidrug resistance by inhibiting transport function of P-glycoprotein (ABCB1) and breast cancer resistance protein (ABCG2) (10). However, the application of apatinib is limited by the emergence of acquired resistance and side effects including hypertension and hand-foot syndrome (11). The combination of two or more anti-tumor agents might be a promising strategy to overcome drug-resistance and increase chemotherapeutic efficacy.

Cordycepin [9-(3-deoxy-β-D-ribofuranosyl) adenine], molecular formula C10H13N5O3 (Supplementary Figure S1), is an active component extracted from Cordyceps militiris (12, 13). The compound ID (CID) of cordycepin in PubChem Compound is 6303. It has been confirmed that cordycepin has anticancer effects on various kinds of cancer cells, including lung cancer (14), colorectal cancer (15), liver cancer (HCC) (16), breast cancer (17), brain cancer (18), and bladder cancer (19) cells *in vitro*. Accumulating evidence implies that cordycepin could promote the phosphorylation of PI3K/Akt and DNA damage, which induce the emergence of reactive oxygen species (ROS) and result in cancer cells apoptosis (20, 21). Additionally, it has also been demonstrated that cordycepin has considerable anticancer effects on drug-resistance NSCLC both *in vitro* and *in vivo*, and its anticancer effect is comparable to currently available targeted therapeutic drugs (22). These studies indicate that cordycepin might be a potential therapeutic option for NSCLC treatment.

The effect of cordycepin combined with targeted therapeutic drugs on NSCLC cells and its underlying mechanisms have not yet been delineated. The aim of this study is to investigate whether cordycepin and apatinib have synergistic anticancer effect on human NSCLC cells, and the potential signaling pathway related to this effect by a series of molecular biology methods.

## Materials and Methods

### Reagents and Chemicals

Cordycepin and apatinib were obtained from Sigma (St. Louis, MO, United States). Cordycepin and apatinib dissolved in physiological saline at a concentration of 20 mM were kept in a refrigerator at −20°C for later use. The RPMI1640 medium and fetal bovine serum (FBS) were from Invitrogen (Carlsbad, CA, United States). The RIPA lysis buffer, proteinase inhibitors and bicinchoninic acid (BCA) protein assay kit were purchased from Beyotime (Nanjing, China).

### Cell Lines and Culture Conditions

The human NSCLC cell lines A549 and PC9 were obtained from the Cell Bank of Chinese Academy of Sciences, and the human NSCLC cell line H1993, human bronchial epithelial (HBE) cell line Bears-2B were from State Key Laboratory of Oncology in South China. All cell lines were incubated at 37°C and 5% CO_2_ in RPMI1640 supplemented with 10% FBS.

### CCK-8 Assay

CCK-8 assay kit (Dojindo Laboratories, Kumamoto, Japan) was applied to test the proliferative inhibition of cordycepin, apatinib, and their combination on the NSCLC cells. A549, PC9, H1993, and Bears-2B cells were plated into 96-well plates (6 × 103/100 μL/well), cultured for 24 h, and treated with different concentrations of cordycepin, apatinib and the combination of both for 48 h. After additionally incubated for 90 min with CCK-8 solution (100 μL/mL), the samples were detected at 450 nm by the microplate spectrophotometer (Thermo Fisher Scientific, Waltham, MA, United States) to measure the optical density (OD). Finally, the SPSS 20.0 software (IBM, Armonk, NY, United States) was applied to calculate the IC50 (50% inhibitory concentration) value.

### Synergy Evaluation

We determined the combination index (CI) through the isobologram analysis on the basis of medium-drug effect analysis. The statistics from the CCK-8 assay were showed as % viability and translated to fraction affected (Fa). Fa ranges from 0 to 1, and Fa = 0 signifies 100% viability, Fa = 1 signifies 0% viability. Then the data were analyzed by the CompuSyn program (Biosoft, Cambridge, United Kingdom). The CI values exhibit the interaction modes between cordycepin and apatinib. CI ranges from 0 to +∞, when CI < 1, it indicates a synergistic effect between the two drugs, when CI = 1, it indicates an additive effect, and when CI > 1, it indicates antagonism.

### Colony Formation Assay

The A549 and PC9 cells were made into single cell suspension and seeded into 6-well plates (400/2 mL/well), with gentle rotation to evenly distribute cells. After maintaining at 37°C and 5% CO_2_ for 24 h, cells were incubated with RPMI1640 containing 10% FBS and different drugs for 2 weeks. When clones are visible in the Petri dish with naked eyes, the cells were carefully washed with PBS twice, and fixed with pure methanol for 20 min. Finally, we stained the cells with 0.5% crystal violet (CV) solution, and the number of cell clones was counted under an inverted optical microscope (Nikon Corporation, Tokyo, Japan).

### Wound Healing Assay

A549 and PC9 cells were plated into 6-well plates (1 × 106 mL/well). When the cell density was about 90% after 24 h, serum-free medium was used to starve the cells for 24 h. Confluent monolayer cells were scratched in a straight line using a 100 μL pipette tip. The exfoliated cells were cleared with PBS (GIBCO) wash for three times. Then the serum free RPMI1640 containing various drugs was used to culture the cells and the cells are allowed to heal the wounds for 48 h. At the same place where cells were scratched, pictures (magnification, 10×) were taken at 0 and 24 h. Ultimately the Adobe Photoshop CS6 software was used to determine the migration length of cells according to the change of wound size.

### Transwell Invasion Assay

A549 and PC9 cells were incubated in serum-free RPMI1640 for 24 h. Subsequently, cells (6 × 104) in 600 μL serum-free medium containing various drugs were plated on the top compartment of transwell filters, which was covered by thin layers of matrigel basement membrane matrix, with 700 μL medium containing 10% FBS in the bottom compartment. The transwell filters were cultured at 37°C with 5% CO_2_ for 48 h. After that, the cells adhering to the bottom membrane were fixated in 4% paraformaldehyde for 30 min, and subsequently dyed with 0.5% CV solution for 15 min at room temperature. Ultimately, the transwell filters were inverted and observed under a microscope (magnification, 100×) for photographic recording and the number of cells on the bottom surface was counted. Five random fields were counted per filter in all groups.

### Apoptosis Assay

After incubation with different groups of treatment for 48 h, the A549 and PC9 cells (5 × 105 mL) were trypsinized before collection for assay. According to the instructions of cell apoptosis detection kit (Beijing 4A Biotech Co., Ltd., Beijing, China), the Annexin V-FITC and propidium iodide (PI) were applied to stain the cells for 30 min under the dark. Finally, the apoptosis rate of cells was detected by ACEABIO NovoCyte flow cytometer (ACEA Biosciences Inc., San Diego, CA, United States) within 1 h after staining. The PI staining levels were analyzed using Novoexpress software and the simulated statistics were tested through Watson (pragmatic) model.

### Cell Cycle Assay

After incubated with different groups of treatment for 48 h, the A549 and PC9 cells (5 × 105 mL) were collected, and suspended in cold 70% ethyl alcohol, incubated at −4°C for 2–24 h. Before detection, cells were cleaned with 1 mL cold PBS again and resuspended in 100 μL RNase, and then incubated at 37°C water bath for 25 min. Then according to the instructions of cell cycle detection kit (Beijing 4A Biotech Co., Ltd., Beijing, China), PI was used to stain the cells for 25 min in the dark at room temperature. At least 50,000 cells were tested for each detection. The amount of cells in each stage of cell cycle was detected by ACEC NovoCyte flow cytometer within 1 h after staining The PI staining levels were analyzed using Novoexpress software and the simulated statistics were tested through Watson (pragmatic) model.

### KEGG Pathway Analysis

Kyoto encyclopedia of genes and genomes (KEGG) is a bioinformatics resource containing abundant data for genome mapping and pathway analysis. KEGG pathway mapping tool was used to obtain the top 15 signaling pathways of cordycepin in KEGG and extract relations between target proteins of cordycepin. The hypergeometric test/Fisher exact test significance threshold *P*-value was set at less than 0.05 (23).

### Western Blot Analysis

After incubation with different groups of treatment for 48 h, the A549 and PC9 cells were harvested and split in RIPA lysis buffer with proteinase inhibitors. The protein concentration of extracts from tested cells were determined by BCA qualitative method. We resolved the extracts in 10% SDS-PAGE (Beyotime, Nanjing, China) and subsequently transferred them to PVDF membranes (Millipore, Bedford, MA, United States). After maintaining in 5% skim milk for 1 h, the PVDF membranes were incubated for 12 h at 4°C in 5% skim milk containing various primary antibodies (VEGFR2, VEGF, PI3K, p-PI3K, Akt, p-Akt 1:1000, caspase-3, Cleaved Caspase-3, Bax, Bcl-2 1:500, and GAPDH 1:5000). All the primary antibodies and horseradish peroxidase (HRP)-conjugated second antibodies were purchased from Cell Signaling Technology. Afterward, the PVDF membranes were washed with tris buffered saline containing 0.1% Tween-20 (TBST) for 10 min, three times, and incubated in HRP-conjugated second antibodies for 1 h. The membranes were washed again with TBST and visualized with ECL regents (Millipore, Bedford, MA, United States). Image J software (NIH image) was used to measure the densitometry of the protein bands.

### Molecular Docking

The crystal structure of PI3K (PDB code: 3APC) was obtained from the Protein Data Bank^1^. The structure of Cordycepin was drawn via ChemDraw, and the energy was minimized with Chem3D. PI3K and Cordycepin were docked with AutoDock 4.2. The protein and ligand were prepared with AutoDock Tools. The docking results were detected using the PyMOL Molecular Graphics System.

### Data Analysis

All experiments were repeated at least three times and the results were showed as mean ± standard deviation (SD). Student’s *t*-test and one-way analysis of variance with the Bonferroni’s correction were applied to analyze the statistics. When *P*-value was less than 0.05, the differences between each group were considered significant.

## Results

### Combination of Cordycepin and Apatinib Synergistically Reduced Proliferation of NSCLC Cells

**FIGURE 1:**
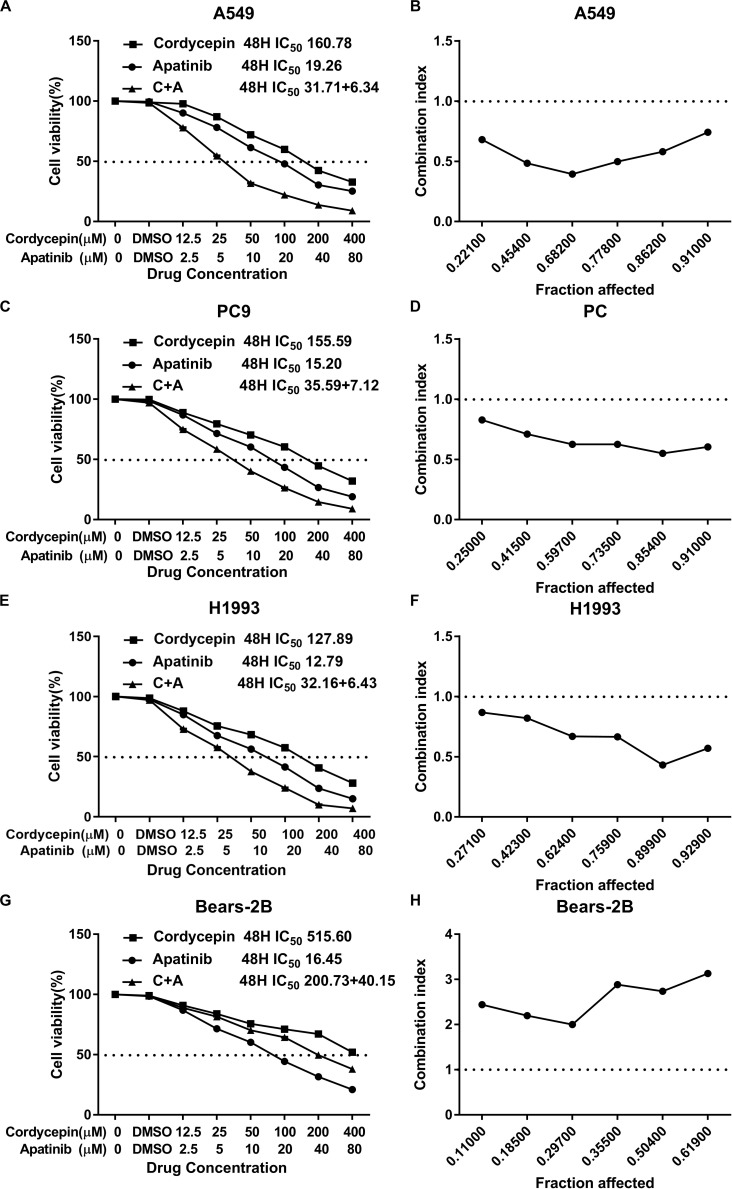
The proliferation inhibitory effect of cordycepin, apatinib, and their combination on the NSCLC cells. Drug concentration-cell viability curves were drawn as the percentage of viable cell based on the cell viability assay **(A,C,E,G)**. The synergistic effects between cordycepin and apatinib were exhibited as Fa-CI plots **(B,D,F,H)**. Data are from three repeated experiments with quadruplicate wells (mean ± SD).

It was found that both cordycepin and apatinib inhibited the growth of NSCLC cells and HBE cells in a dosage- and time-dependent manner. Among the tested cells, Bear-2B cells showed a highest IC50 value of using cordycepin, indicating that Bear-2B cells have a weaker susceptibility to cordycepin compared with the NSCLC cells (Figure 1).

**FIGURE 2:**
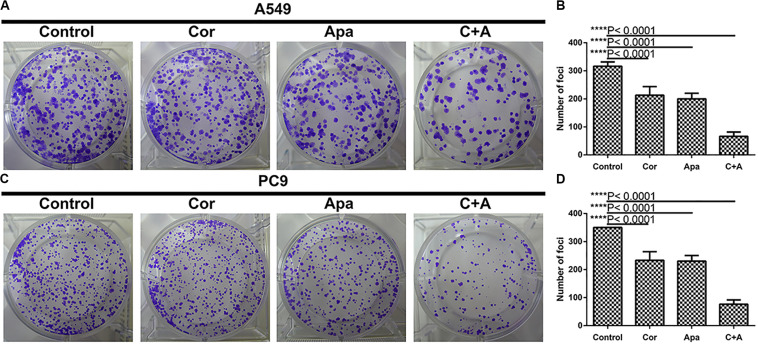
Cordycepin and apatinib suppressed colony formation of A549 and PC9 cells. **(A,C)** Cells were treated with 150 μM of cordycepin, 15 μM of apatinib or 30 μM of cordycepin, and 6 μM of apatinib in combination; **(B,D)** statistics of **(A)** and **(C)**. ****P < 0.0001 versus the control group.

Based on the calculated IC50 values of cordycepin and apatinib, cells in the combinational treatment group were treated with the combinations of cordycepin and apatinib in a fixed molar ratio of 5:1. The NSCLC cells treated with combinational drugs showed a stronger suppressive effect on cell proliferation compared with cells treated with each of the single drug (Figure 1). As showed in Figure 2, the combination group displayed the least number of clones among the tested groups.

Whatever the Fa value was, the CI values of A549, PC9, and H1993 cells were <1, which meant synergism, and the CI value of Bear-2B cells was >1, which meant antagonism. Table 1 summarized the CI values and the concentrations of individual drugs in combination at 50% Fa.

**TABLE 1:** Summary of CI value and the concentration of separate drugs in combination at 50% Fa.

Drug combination	Fa = 0.5			
	A549	PC9	H1993	Bears-2B
**Apa + Cor**				
CI	0.52641	0.69300	0.74313	2.78935
Apa (μM)	6.47237	7.15023	6.37445	39.9515
Cor (μM)	32.3618	35.7511	31.8723	199.758

### Combination of Cordycepin and Apatinib Synergistically Restrained the Migration and Invasion Capacity of NSCLC Cells

**FIGURE 3:**
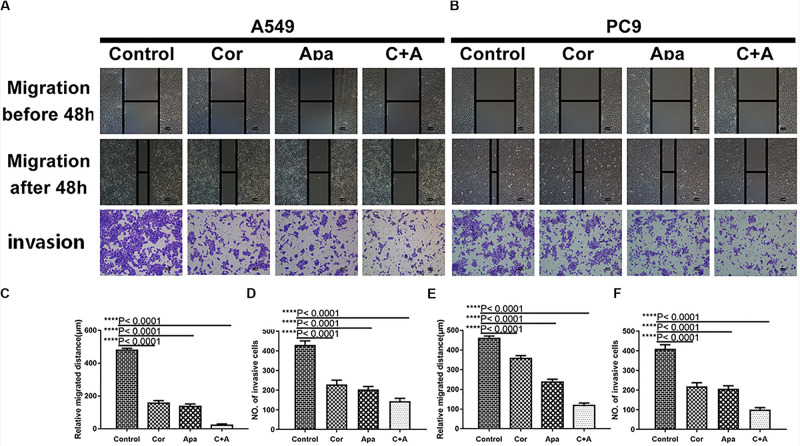
Cordycepin and apatinib suppressed migration and invasion ability of A549 and PC9 cells. Typical images of wound healing and transwell assay **(A,B)** after 48 h treatment with 150 μM of cordycepin, 15 μM of apatinib and 30 μM of cordycepin, and 6 μM of apatinib in combination. Histograms show the average migration distance **(C,E)**, and the amount of invasive cells **(D,F)**. All data are shown as the mean ± SD of three independent experiments. ****P < 0.0001 versus the control group (magnification, ×100; scale bars, 100 μm).

To investigate whether cordycepin and apatinib affect the migration and invasion ability of NSCLC cells, wound healing assay and transwell assay were performed on the A549 and PC9 cells. It was showed in wound healing assays that both cells migrated the least distance in the combinational treatment group after 48 h, and the combination treatment exhibited the least number of invasive cells in transwell assay (Figure 3).

### Combination of Cordycepin and Apatinib Induced NSCLC Cell Apoptosis by Altering Cell Cycle

**FIGURE 4:**
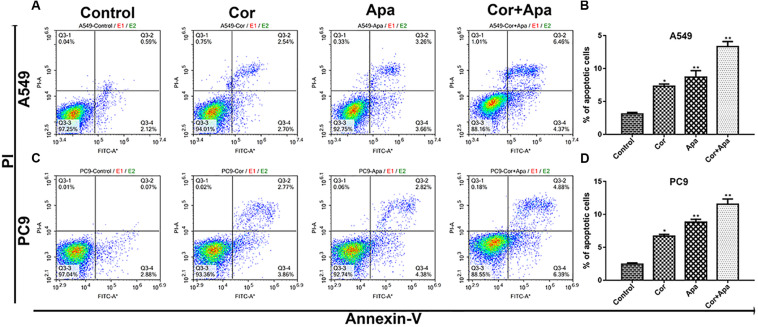
Effect of cordycepin, apatinib alone and both in combination on cell cycle. The percentage of cells in G1, S, or G2/M phase in A549 **(A)** or PC9 cells **(C)** treated with 150 μM of cordycepin, 15 μM of apatinib or 30 μM of cordycepin, and 6 μM of apatinib in combination for 48 h. Data represent the cell population in each phase of A549 **(B)** and PC9 **(D)**. All data are shown as the mean ± SD of three independent experiments. *P < 0.05, **P < 0.01 versus the control group.

Since the co-treatment of cordycepin and apatinib increased cell death of NSCLC cells, we examined the change of cell cycles and the apoptosis of A549 and PC9 cells by Flow cytometry. It was showed in Figure 4 that both cordycepin and apatinib primarily enhanced the G1 phase cell population compared with the untreated group, and the combined treatment achieved the most significant effect.

**FIGURE 5:**
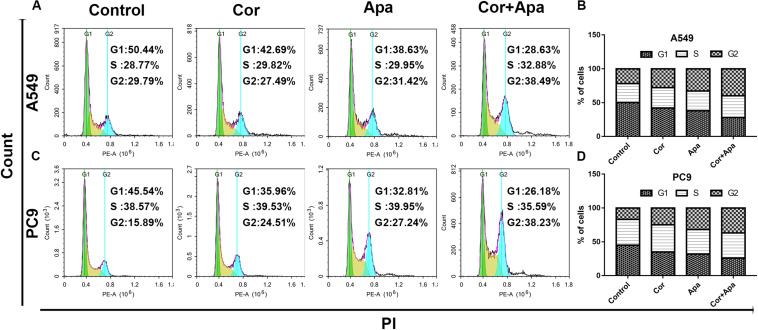
Effect of cordycepin and apatinib alone and in combination on cell apoptosis. Representative profiles showing apoptosis in A549 **(A)** and PC9 **(C)** cells treated with 150 μM of cordycepin, 15 μM of apatinib and 30 μM of cordycepin, and 6 μM of apatinib in combination for 48 h. Histograms represent the average apoptosis rate of A549 **(B)** and PC9 **(D)**. All data are shown as the mean ± SD of three independent experiments.

As showed in Figure 5, both individual drug treatment and combinational drug treatment led to early and late apoptosis of NSCLC cells. In addition, the combinational drug treatment induced apoptosis more effectively in comparison with individual drug treatment.

### Combination of Cordycepin and Apatinib Down-Regulated Protein Molecules in the VEGF/PI3K/Akt Signaling Pathway in NSCLC Cells

**FIGURE 6:**
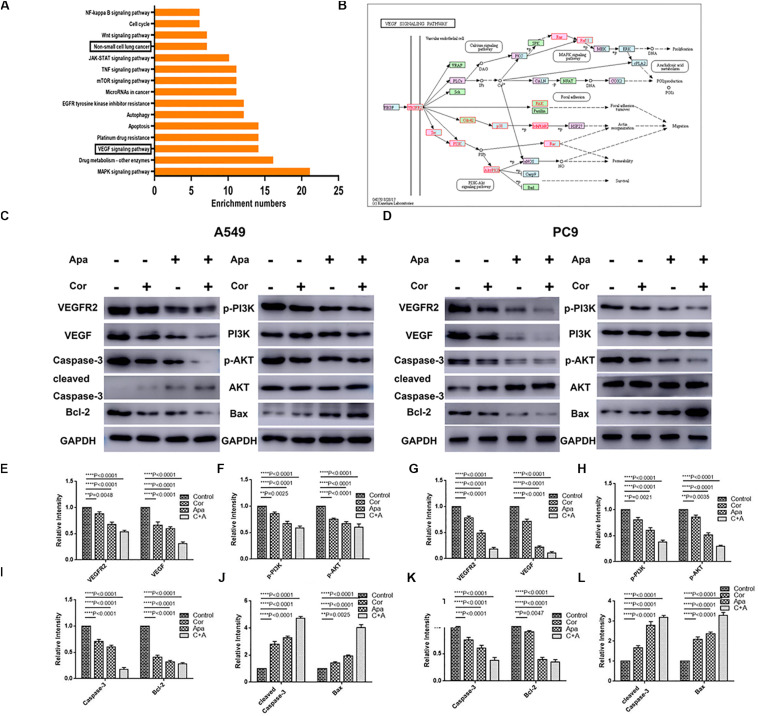
KEGG pathway analysis of cordycepin and the VEGF signaling pathway. **(A)** The top 15 signaling pathways affected by cordycepin in KEGG pathway analysis. **(B)** The VEGF signaling pathway and the potential target protein (red) of cordycepin. **(C,D)** Suppressive effect of cordycepin, apatinib or their combination on VEGF signaling pathway in A549 **(C)** and PC9 **(D)** cells. Figures are the protein expression of Bax, Bcl-2, Cleaved Caspase-3, Caspase-3, p-Akt, Akt, p-PI3K, PI3K, VEGFR2, VEGF, and GAPDH of A549 and PC9 cells treated with 150 μM of cordycepin, 15 μM of apatinib alone or 30 μM of cordycepin, and 6 μM of apatinib in combination for 48 h. **(E–H)** Relative intensity of protein on VEGF signaling pathway in A549. **(I–L)** Relative intensity of protein on VEGF signaling pathway in PC9. All data are shown as the mean ± SD of three independent experiments. **P < 0.01, ***P < 0.001, or ****P < 0.0001 versus the control group.

The top 15 signaling pathways related to treatment of cordycepin were revealed through KEGG pathway analysis (Figure 6A). Among the 15 pathways, vascular endothelial growth factor (VEGF) signaling pathway was primarily affected by cordycepin. The potential target protein (red) of cordycepin was further explored (Figure 6B).

Given the fact that both cordycepin and apatinib significantly induced the apoptosis of NSCLC cells, we used western blotting to evaluate the expression of related proteins in apoptotic signaling pathways including VEGFR2, VEGF, p-PI3K, PI3K, Akt, p-Akt, Caspase-3, Cleaved Caspase-3, Bax, and Bcl-2. Compared with the untreated group (control group), the expression of VEGFR2, VEGF, p-Akt, p-PI3K, Bcl-2, and Caspase-3 decreased, and the levels of Bax, Cleaved Caspase-3 increased in both individual drug and combinational drug treatment groups, while the total levels of Akt, PI3K remained unchanged (Figures 6C–L). The combinational drug treatment group exhibited the most notable difference among all groups.

### Molecular Docking Study and ADMET Prediction

**FIGURE 7:**
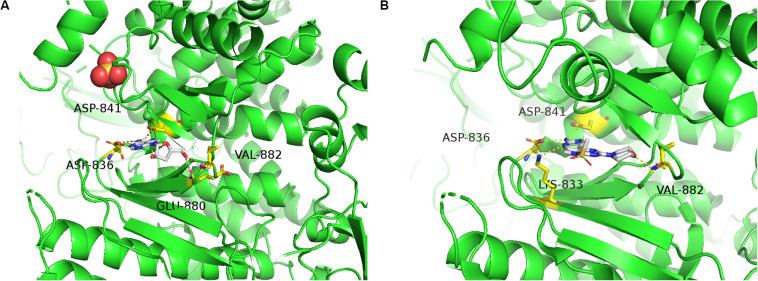
Molecular docking study and ADMET prediction of Cordycepin with PI3K. **(A)** Predicted binding model of Cordycepin with PI3K (Binding Energy is –6.69). Hydrogen bonds are shown as red dashed lines. **(B)** X-ray crystal structure of PI3K inhibitor bound to the active pocket of PI3K showing the “propeller shape” and the key hinge interactions with ASP836, ASP841, LYS833, and VAL882 (PDB code 3APC), visualized in PyMOL.

To predict the possible binding mode of Cordycepin with PI3K, we performed a molecular docking study. The crystal structure of PI3K (PDB code: 3APC) was downloaded from the Protein Data Bank for the docking calculations. As shown in Figure 7A Cordycepin interacts with residues ASP836, ASP841, GLU880, and VAL882 through hydrogen bond (H-bond) interactions in the active pocket of PI3K (Binding Energy is −6.69), 3 H-bonds are similar to PI3K inhibitor (Phosphatidylinositol-4,5-bisphosphate 3-kinase catalytic subunit gamma isoform) via X-ray crystallographic analysis (Figure 7B). Taken together, the above results demonstrated that Cordycepin occupies the binding site of PI3K and displays vital molecular docking interactions with contiguous amino acids.

## Discussion

Apatinib, as a small molecule targeted anti-angiogenesis, has a strong inhibitory effect on VEGFR-2 signaling in lung, colorectal and gastric cancers (24). However, the emerging acquired drug resistance and side effects have limited its application, which also implicated the limitation of monotherapy.

An increasing number of studies have revealed the significant effect of cordycepin on inhibiting proliferation of various cancer cells (14, 16, 22). The inhibitory effect of cordycepin was much weaker than that of apatinib when used as a single drug treatment. In this study, it was demonstrated that cordycepin can suppress the proliferation of NSCLC cells in a dosage- and time-dependent manner. And different from the three NSCLC cell lines, Bears-2B cells showed a lower sensitivity to the treatment with combinational drugs, which indicated that cordycepin could enhance the sensitivity of apatinib on NSCLC cells and reduce its toxicity on normal HBE cells.

Further study revealed that co-treatment of cordycepin and apatinib was able to synergistically reduce cell proliferation, inhibit cell migration and invasion. Co-treatment of cordycepin and apatinib also lead to cell-cycle arrest and apoptosis in A549 and PC9 cells. It was found that the potential mechanisms of apoptosis caused by combinational use of cordycepin and apatinib in NSCLC cells may be related to suppression on the activity of VEGF/PI3K/Akt signaling pathway. Therefore, cordycepin may increase the sensitivity of apatinib on NSCLC, and attenuate the side effects by minimizing the dose of apatinib.

Our results suggested that both cordycepin and apatinib increased the G1 phase cell population in NSCLC cells, which was consistent with other studies. It has been demonstrated in Zheng’s study that cordycepin could increase G1 phase arrest and finally lead to induction of apoptosis in H1975 cells (25). Similar results were also found in human colorectal cancer cells, in which the cell cycle progression of SW480 and SW620 cells was arrested at the G1 phase after the addition of cordycepin (26). However, there were controversial results on the effect of cordycepin on the distribution of cell cycle. Cho’s study reported that the proportion of the sub-G1 phases of cells treated with cordycepin and cells in control group were 15.9 ± 0.9% and 5.4% ± 0.9%, respectively, after 24 h of treatment, which indicated that cordycepin promoted apoptosis through accumulation of sub-G1 in A549 cisplatin-resistance lung cancer cells (27). And Lee’s research revealed that cordycepin enhanced sub-G1 and G2/M phase arrest of HT-29 cells at the concentration of 100 μM, while cordycepin at 200 and 400 μM enhanced G1 phase arrest (28). These studies suggested that low dose of cordycepin might lead to cell cycle arrest at sub-G1 and G2/M phases, while high dose of cordycepin probably induce cell cycle arrest at G1 phase in NSCLC cells, which needs to be further explored.

Through KEGG pathway analysis, significant difference was found in VEGF signaling pathway. As crucial regulators of angiogenesis, VEGFs consist of five secreted proteins in mammals such as VEGF-A, B, C, D, and placental growth factor (PLGF). These VEGFs have different binding affinities to three kinase receptors (VEGFR-1, 2, 3) (29, 30). VEGFR-2 binds to VEGF-A, acting as the major mediator of mitosis, proliferation and survival in endothelial cells (31). Phosphoinositide 3-kinase (PI3K)/protein kinase B (AKT) pathway can be activated by growth factors and angiogenesis inducers such as VEGF and angiopoietins (32). A plenty of studies have demonstrated that PI3K/Akt was the main downstream cellular pathway mediating the biological effects of VEGF/VEGFR-2 (33–35), the VEGF/PI3K/Akt signaling pathway plays an significant role in various cellular processes including proliferation, migration, invasion, and metastasis (34, 36).

Vascular endothelial growth factor receptor-2 tyrosine kinase inhibitors such as apatinib might block the combination of VEGF and VEGFR-2, leading to suppressing the downstream PI3K/Akt signaling pathway by interrupting the phosphorylation of PI3K and Akt. Meanwhile, the proapoptotic proteins including Bax, Bad and caspase families could not been phosphorylated by the inactivated Akt, finally causing the apoptosis of cells (5, 37).

In our study, the result of Western blotting showed the low expression levels of VEGFR2, VEGF, p-Akt, p-PI3K, Bcl-2, Caspase-3, and the high expression levels of Bax, and Cleaved Caspase-3. The levels of Akt and PI3K proteins remained almost unchanged after drug treatment. The difference of protein expression in cells treated with combinational drugs was more significant than individual drug groups. The result of molecular docking indicated that Cordycepin occupies the binding site of PI3K and displays vital molecular docking interactions with contiguous amino acids. Recent researches have reported that cordycepin could induce apoptosis through regulating PI3K/Akt signaling pathway in multiple tumor cells such as SGC 7901 cells (20), Leydig tumor cell (38), and LNCaP human prostate carcinoma cells (39), that were consistent with our results. Since apatinib acts as a selective VEGFR-2 tyrosine kinase inhibitor, cordycepin and apatinib probably induce cell apoptosis via down-regulating the VEGF/PI3K/Akt signaling pathway.

There are some defects of the present study. The toxicity and efficacy of this combination in animal is not known, since animal study with xenografts were not conducted.

## Conclusion

In conclusion, the current study revealed that cordycepin in combination with apatinib leads to increased cell apoptosis, decreased cell proliferation, migration, and invasion of NSCLC cells through down-regulating VEGF/PI3K/Akt signaling pathway. This study provides a rational of using cordycepin in combination with apatinib as a promising strategy for the treatment of NSCLC.

## Data Availability Statement

The raw data supporting the conclusions of this article will be made available by the authors, without undue reservation.

## Author Contributions

LS, Z-SC, and LL: conceptualization. XL, LT, and WG: methodology. JW and Z-XW: software. LT, HD, JZ, and HC: validation. LS and XL: formal analysis. LT and LS: writing – original draft preparation. Z-SC, Z-XW, and LL: writing – original and editing. LL and Z-SC: supervision. LS, LL, and XL: funding acquisition. All authors have read and agreed to the published version of the manuscript.

## Conflict of Interest

The authors declare that the research was conducted in the absence of any commercial or financial relationships that could be construed as a potential conflict of interest.

## Footnotes

1 https://www.rcsb.org/

## Supplementary Material

FIGURE S1 The 2D structure of Cordycepin **(A)** and Apatinib **(B)** (from PubChem compound http://pubchem.ncbi.nlm.nih.gov/).
