# Supplementary figures and images for: Combination of Cordycepin and Apatinib Synergistically Inhibits NSCLC Cells by Down-Regulating VEGF/PI3K/Akt Signaling Pathway
Source: Front Oncol. 2020 Sep 7;10:1732. doi: 10.3389/fonc.2020.01732 (PMC7505117; doi:10.3389/fonc.2020.01732)

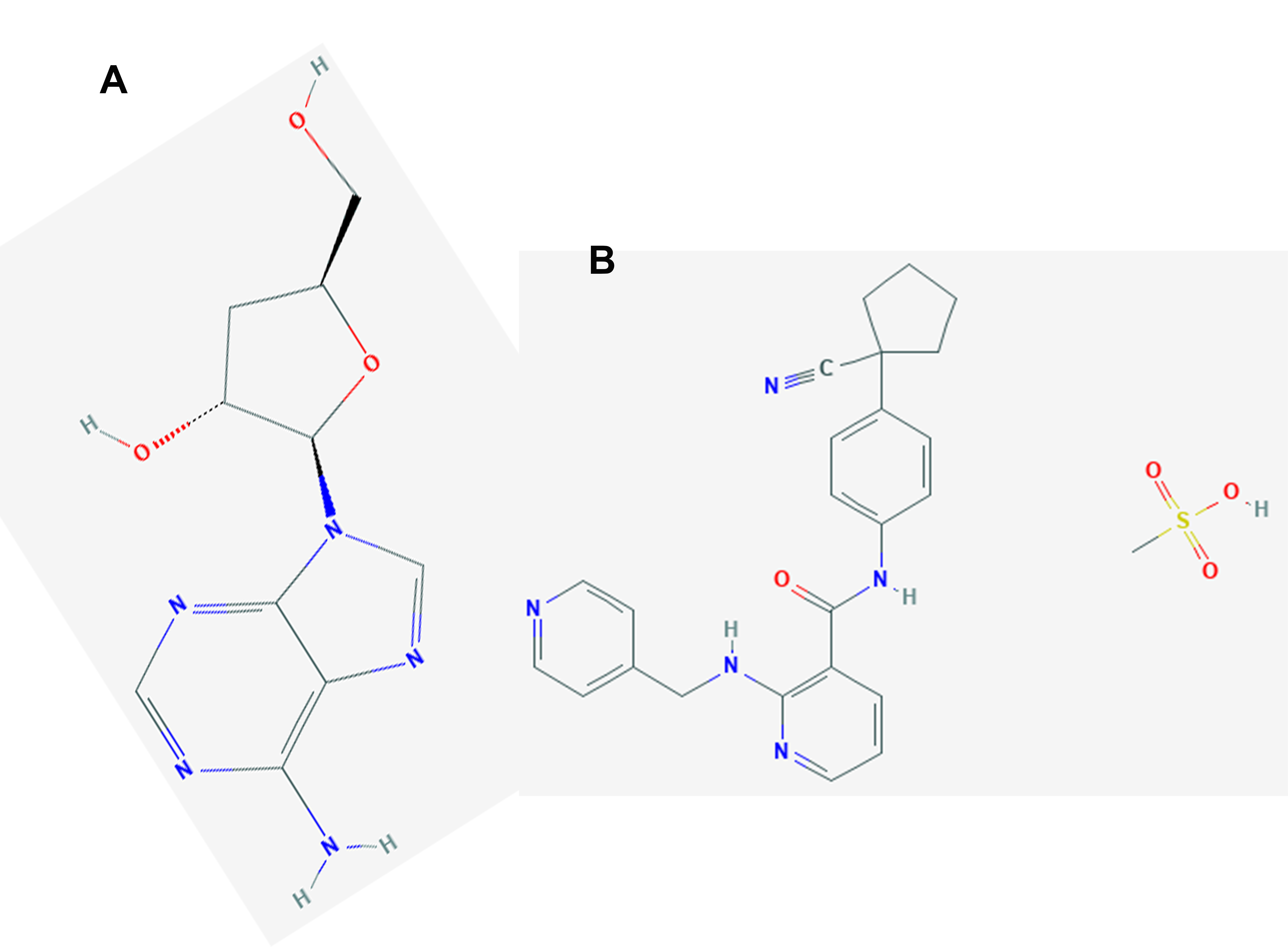

Supplement: FIGURE S1 — The 2D structure of Cordycepin (A) and Apatinib (B) (from PubChem compound http://pubchem.ncbi.nlm.nih.gov/). [file Image_1.TIF]
